# Using agro-ecological zones to improve the representation of a multi-environment trial of soybean varieties

**DOI:** 10.3389/fpls.2024.1310461

**Published:** 2024-03-25

**Authors:** Catherine Gilbert, Nicolas Martin

**Affiliations:** University of Illinois at Urbana-Champaign, Department of Crop Sciences, Urbana, IL, United States

**Keywords:** agro-ecological zones, multi-environment trials, environmental representation, soybean adaptation, soybean maturity groups, target population of environments, spatial clustering, bioclimatic variation

## Abstract

This research introduces a novel framework for enhancing soybean cultivation in North America by categorizing growing environments into distinct ecological and maturity-based zones. Using an integrated analysis of long-term climatic data and records of soybean varietal trials, this research generates a zonal environmental characterization which captures major components of the growing environment which affect the range of adaptation of soybean varieties. These findings have immediate applications for optimizing multi-environment soybean trials. This characterization allows breeders to assess the environmental representation of a multi-environmental trial of soybean varieties, and to strategize the distribution of testing and the placement of test sites accordingly. This application is demonstrated with a historical scenario of a soybean multi-environment trial, using two resource allocation models: one targeted towards improving the general adaptation of soybean varieties, which focuses on widely cultivated areas, and one targeted towards specific adaptation, which captures diverse environmental conditions. Ultimately, the study aims to improve the efficiency and impact of soybean breeding programs, leading to the development of cultivars resilient to variable and changing climates.

## 1 Introduction

Soybean breeders uncover genotype x environment interactions (GEIs) using multi-environment trials (METs). METs evaluate the performance of the population of genotypes across a network of test sites intended to represent the target population of environments (TPE), or the range of future environments in which the varieties are expected to be grown (Yan et al., 2011; Oakey et al., 2016; Crespo-Herrera et al., 2021). The intention is that the test sites will recreate the GEIs of the TPE, so that breeders can use the performance of a variety at one or more test sites to predict its performance in environmentally similar locations within the TPE (Chenu, 2015).

An ideal network of test sites should represent the TPE as closely as possible, to best recreate the GEIs of those environments (Allen et al., 1978). The test sites in total should capture enough of the environmental variation of the TPE that adapted varieties can be developed for those conditions, and that the performance of new varieties can be predicted to the full range of target growing environments. Breeders selecting for specific adaptation might choose to prioritize sites that represent unique regional variation, while breeders selecting for general adaptation might choose to prioritize sites that represent the environments where the crop is most frequently grown (Hamblin et al., 1980; Annicchiarico et al., 2005; Rattalino Edreira et al., 2018). Because METs can only operate a finite number of test sites, the set of sites should represent the conditions of the TPE as efficiently as possible (Cooper et al., 2022). Test sites should not be so environmentally similar that the results from two or more sites are redundant or that a particular range of environments becomes over-represented (Yan et al., 2011).

By optimizing the distribution of MET resources with respect to environmental representation, soybean breeders may be able to better represent the variation of the TPE and more efficiently develop adapted soybean varieties (Hyman et al., 2013; Xu et al., 2017; González-Barrios et al., 2019; Resende et al., 2021). The increasing availability of high-resolution bioclimatic data provides new opportunities to characterize the environmental variation of the soybean TPE in dimensions that are relevant to the adaptation of soybean varieties (Hyman et al., 2013).

Spatial bioclimatic variation is a major component of the environment that affects soybean adaptation. The relative performance of soybean cultivars between locations is strongly related to parameters of temperature, precipitation, and terrain which vary continuously across the growing environment (Xu, 2016; Gao et al., 2020; Zhang et al., 2020; Arya et al., 2021).

Agro-ecoregionalization is a method of characterizing environmental variation across a TPE by subdividing the geographical area into regions which are similar in terms of ecological characteristics which are relevant to a crop’s performance. The resulting agro-ecological zones (AEZs) represent more environmentally homogenous subregions of the TPE (Williams et al., 2008). Previous studies have used AEZs derived from the multivariate clustering of environmental data to interpret the environmental variation of a target area, and to inform research and production decisions (Castrignanò et al., 2010; Boitt et al., 2014; Costantini et al., 2016; Alabi et al., 2019; Setimela et al., 2005; Williams et al., 2008; Zhang et al., 2012). Here, we identify a set of distinct, agronomically significant variables and use them to cluster the soybean TPE into AEZs which represent the bioclimatic variation of the growing environment.

A second component of the environment that affects soybean adaptation is maturity. Maturity is the most important agronomic characteristic for determining a soybean’s adaptation to a particular location (Bandillo et al., 2017). Because of the photoperiod-sensitive responses of soybeans, each particular variety of soybean is restricted to a relatively narrow range of latitudes (Watanabe et al., 2012). The optimum maturity groups of the growing region, or the maturities of soybeans best suited to any location, must therefore be known to effectively breed and recommend soybean varieties (Zhang et al., 2007).

Soybean maturity groups should be characterized and re-characterized with new data to account for climate change or possible drift in how designations are defined (Mourtzinis and Conley, 2017; Ersoz et al., 2020). Recent soybean variety trials are reliable source of data for this task because they are conducted under optimum planting conditions using the best management practices for soybean within the region (Zhang et al., 2007). Here we create an updated map of maturities using data from the Northern Uniform Soybean Tests (NUSTs), modeling the maturity designations of each variety, then finding the best performing maturity designations across the TPE. We then model the best performing maturities across the conditions of the TPE from the best performing maturities at the conditions of each site and divide the TPE into maturity groups (MGs) indicating the optimum maturities for each region.

The NUSTs are a major MET of public North American soybean varieties conducted at test sites across the northern United States and southern Canada. As with most public METs, the resources in the NUSTs have not been distributed according to a formal strategy of environmental representation (Aaron Lorenz, personal communication, March 23, 2023).

The objective of this study was to use long-term agroclimatic data to classify the NUST TPE, the area of soybean cultivation in the United States and Canada, into zones representing components of the growing environment that limit soybean adaptation. Agro-ecological zones (AEZs) were used to capture the spatial bioclimatic variation of the TPE, and maturity groups (MGs) were used to capture optimum maturities. We then used these characterizations to suggest how NUST testing resources might hypothetically be distributed to improve the test network’s representation of environmental variation within the TPE. We provided two potential distribution strategies: one targeted at general adaptation which prioritized representation of areas where soybean is most frequently grown, and one targeted at specialist adaptation which prioritized representation of the full environmental variation of the soybean TPE.

## 2 Materials and methods

This research used the records of the Northern Region Uniform Soybean Tests (NUSTs), an MET of public soybean varieties conducted yearly across the northern United States and Canada. The data from these trials are available as printed reports (USDA ARS, 2022) and through the SoyBase database (Brown et al., 2021). We chose to use records of the uniform tests specifically and exclude records from preliminary variety tests. These records span the years 1989 to 2021 and contain the results of tests performed on 9316 varieties at 233 test sites. Raster manipulation and analysis for this research was performed using the terra package in R (Hijmans et al., 2023).

### 2.1 Defining agro-ecological zones

We began by defining the extent of the TPE. Because this research uses data from a North American soybean varietal trial, the extent of the TPE would be the area in North America in which soybean is typically grown. Two rasters of soybean cultivation frequency were used to define this area, one of cultivation in the United States from 2007 – 2022 at approximately 0.054 degrees of resolution (USDA NASS, 2022), and one of Canada from 2009 – 2022 at approximately 0.003 degrees of resolution (AAFC, 2022). The Canadian raster was resampled to the higher resolution, and the rasters were combined. The final cultivation raster was then filtered to areas where soybean had been grown for at least 15% of the years recorded.

We then obtained long-term climatic data for the area of TPE. This information was sourced from the WorldClim bioclimatic rasters, which contain the averages of nineteen bioclimatic variables as measured from 1970 – 2000 interpolated to approximately 0.042 degrees of resolution (Fick and Hijmans, 2017). On the advice of breeders, we also chose to include the elevation raster which was used to generate the WorldClim rasters. The WorldClim elevation raster was derived from SRTM data and had a matching resolution of approximately 0.042 degrees (Fick and Hijmans, 2017).

**Figure 1:**
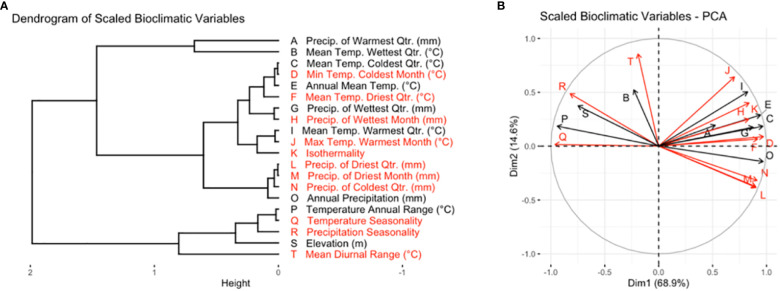
Variables in black were included in the subset of bioclimatic variables used for cluster analysis and the creation of AEZs, while variables in red were dropped. **(A)** Dendrogram of scaled bioclimatic variables for the area of soybean cultivation in the United States and Canada. **(B)** PCA variable correlation plot of scaled bioclimatic variables for the area of soybean cultivation in the United States and Canada. The letters on each ray correspond to the bioclimatic variables as they are labeled in **(A)**.

The full set of rasters was masked to the previously defined boundaries of the TPE. Because many of the temperature and precipitation variables were highly correlated, a representative subset of bioclimatic variables was selected from the full set of variables (**Figure 1A**). Reducing the number of explanatory variables also had the advantage of facilitating the interpretation of patterns of environmental variation within the TPE, and for modeling the classification of AEZs.

We aimed to choose a set of variables that were distinct in effect, associated with environmental variation within the TPE, and relevant to soybean cultivation. Principal component analysis (PCA) was used to understand the correlation between the variables and identify distinct variables which drove environmental variation within the TPE (**Figure 1B**). We ensured that the set of variables chosen was relevant to soybean cultivation by seeking out breeder feedback. A preliminary version of this research was presented at the 2022 ASA-CSSA-SSSA conference and 2023 Soybean Breeders’ Workshop, where the authors spoke with breeders to learn which bioclimatic variables they felt had the greatest effect on soybean cultivation.

The final variables chosen were annual mean temperature, temperature annual range, mean temperature of the wettest quarter, mean temperature of the warmest quarter, mean temperature of the coldest quarter, annual precipitation, wettest quarter precipitation, warmest quarter precipitation, and elevation.

We created AEZs by clustering the subset of environmental variables. The number of clusters to use was determined with the cubic clustering criterion, which estimated the optimum number of clusters as thirteen. The nine bioclimatic variables were scaled and clustered with k-means clustering into thirteen regions. These became the initial set of AEZs.

In the initial set of AEZs, Illinois and Indiana were almost entirely covered by a single AEZ. This AEZ would be largest of any in the set, with the greatest cultivation, and represent the most productive land for growing soybeans. After receiving feedback from NUST breeders, we split this AEZ in two using soil properties.

Soil data for the TPE was obtained from the SoilGrids database (ISRIC, 2023). Rasters of soil properties were downloaded from the ISRIC WebDAV server using a script provided by Dr. José Safanelli (Safanelli, 2022). The rasters we obtained were bulk organic density, cation exchange capacity, clay, nitrogen, soil pH, sand, silt, and soil organic carbon, collected at a depth of 15 – 30 cm and interpolated to approximately 0.054 degrees of resolution.

The most agronomically relevant division within the original zone was between the highly fertile mollisols and relatively less fertile alfisols. Soils of the former order have a greater soil organic carbon (SOC) content, which contributes to their productivity (Guo et al., 2006). Because of this distinction, we decided to split the zone by SOC content. Cells in the top quartile of SOC content were assigned to one AEZ, while the remainder three quarters of the cells were assigned to another. This resulted in the AEZs that would be labeled 8 and 9.

Once the zone was split, we numbered the AEZs from north to south to obtain the full set of agro-ecological zones. We reviewed the final AEZ map with soybean breeders to confirm that the AEZs that we created were meaningful and relevant to their breeding programs.

### 2.2 Defining maturity groups

Maturity groups describe the adaptation of cultivar maturities. Each maturity group is equivalent to a range of maturity designations which are best adapted to that region, or the maturities which are expected to return the highest yields when grown over a full season. Maturity groups are a categorical representation of a continuous gradient of variation. The “optimum maturity” of any location is its best adapted maturity, or the specific cultivar maturity which maximizes yield in that environment (Mourtzinis and Conley, 2017). Using records of performance and maturity across MET sites, one can identify best adapted maturities, predict optimum maturity across the TPE, and delineate maturity groups (Zhang et al., 2007; Mourtzinis and Conley, 2017; Di Mauro et al., 2022). For this analysis, the NUST records were combined with environmental data and used to predict optimum soybean maturities across the TPE. This data was used to generate a raster of maturity groups which could be combined with the previously generated AEZs.

We began by converting the NUST maturity records to a continuous scale. Uniform check designations were formatted as from 00 to IV and as “Early”, no description, or “Late”. These categorical MG designations were converted to quantitative relative maturity (RM) designations by taking the value of the MG designation as a roman numeral and adjusting early and late checks by - 0.5 or + 0.5 respectively. For example: a cultivar with a designation of MG III became RM 3, a cultivar with a designation of Early-MG IV became (4 - 0.5 =) RM 3.5, and a designation of Late-MG II became (2 + 0.5 =) RM 2.5. MG 00, which is shorter than MG 0, was equivalent to RM -1.

Within the NUSTs, the only cultivars with given maturity designations are maturity checks. This creates an issue when modeling optimum maturity from the best-adapted maturities, as most cultivars would not have associated maturity designations. To expand the scope of our training data, the maturity designations associated with records of non-check cultivars were predicted from the date at which the plants reached maturity relative to checks in the same year and location. A mixed effects model of RM designation from day of maturity for each location in each year was trained on check cultivars with converted RM designations and used to predict the RM designations for records from undesignated cultivars (Equation 1). The predicted maturity designations were bounded to a range of -2 to 5, equivalent to a half MG past the most extreme checks within the test system at Early-MG 00 (= RM -1.5) and Late-MG IV (= RM 4.5) respectively.


(1) $$RM_{ijk}=\alpha \;+\;DM_{i}\;+\;L_{k}\;+\;{\left(Y|L\right)}_{jk}+\;\epsilon_{ijk}$$

Equation 1 The mixed effects model is used to predict the relative maturity of a cultivar *i* from the cultivar’s day of maturity, given the test location and ear of the trial. Where *RM_i_* = relative maturity for cultivar *i*, *DM* = day of maturity, *Y* | *L_j_* = random effect of year *j* within location *k*, *L_k_* = random effect of location *k.*

After each cultivar record was associated with an RM designation, the NUST records were filtered, for each year and location, to records in the ninetieth percentile of yield. These records of best performing varieties were understood as representing the varieties best adapted to each site, and the RM designations associated with these records were used as “best-adapted maturities.” The predicted best adapted maturity at each location within the TPE, or the maturity designation that would maximize yield in that environment, would be the optimum maturity of that location.

With the best-adapted maturities of each site determined, environmental data was used to predict optimum maturity across the range of the TPE. Soybean maturation is a function of photoperiod, or daylength, and temperature, so these environmental variables were used to predict the best adapted maturity at each location (Garner and Allard, 1930; Major et al., 1975). Elevation was also incorporated as a necessary component of location when modeling maturity (Mourtzinis and Conley, 2017; Marcillo et al., 2021).

The geosphere package’s daylength function was used to calculate the mean photoperiod in hours between May 20^th^ and September 7^th^ for latitudes within the area of soybean cultivation (Forsythe et al., 1995). May 20^th^ and September 7^th^ were chosen as start and end dates for determining mean photoperiod because these are the median dates of planting and harvest for the trial dataset. The mean photoperiod at each latitude within the TPE was converted to a raster with approximately 0.054 degrees of resolution. Temperature and elevation data were obtained from the rasters of annual mean temperature and elevation included with the WorldClim bioclimatic rasters.

The rasters of mean photoperiod, mean annual temperature, and elevation were used as predictors in a generalized linear model to predict optimum maturity at each point in the soybean TPE (Equation 2).


(2) $${\hat{RM}}_{k}=2.36-1.56{\left(MPP\right)}_{k}-9.99{\left(MAT\right)}_{k}-8.48{\left(ELV\right)}_{k}+9.71{\left(MPP\times MAT\right)}_{k}+\;8.50{\left(MPP\times ELV\right)}_{k}+5.19{\left(MAT\times ELV\right)}_{k}-5.18{\left(MPP\times MAT\times ELV\right)}_{k}$$

Equation 2. The generalized linear regression model used to predict the maturity designation, or the best adapted maturity, of a new location *k*. *RM*= relative maturity designation of the location, *MPP* = mean photoperiod, *MAT* = mean annual temperature, *ELV*= elevation.

The predicted RM values of each cell were rounded to the nearest integer and converted to categorical MG designations. As the reverse of the previous conversion, numbers became roman numerals, with -1 again equivalent to “00.” This effectively classified the continuous gradient of RM values into bands of categorical MGs, with divisions between MGs at of -0.5 to +0.5 of each whole RM value. These were the final maturity groups.

### 2.3 Defining AEZ-MGs

After obtaining the MG map, we intersected the AEZ and MG maps to create AEZ-MG classifications which capture the spatial environmental variation and optimum maturities of the TPE.

Because these AEZ-MGs were intended to be used as a means of interpreting environmental variation within the TPE for the distribution of MET resources, we avoided creating very minor AEZ-MGs which would not carry enough significance to be relevant to trial design. We edited the boundaries of the AEZs to remove most AEZ-MGs less than 500,000 acres^2^ in size and classified that land to the largest bordering AEZ, when available. This resulted in the removal of AEZ-MGs 10-II, 1-I, 7-IV+, 2-I, 7-I, 4-0, 5-0, and 9-II. None of these AEZ-MGs contained a testing site with records. When these AEZ-MGs were included in a run of our later analysis, none represented enough environmental variation or relative cultivation to be allocated testing or affect recommendations.

Each NUST test site was given an AEZ and MG classification. The MG classification of a site was determined by which maturity group the site fell within. AEZ classification was two-step, to account for sites outside the TPE, and therefore outside the boundaries of the AEZs. Sites within the boundaries of an AEZ were classified as belonging to that AEZ. Sites outside the boundaries of the AEZs, i.e., in areas where soybean was grown for less than 15% of the years recorded, were given AEZ classifications using a Least-Discriminant Analysis model trained from the bioclimatic variables which were used to generate the AEZs and the associated AEZ classifications.

### 2.4 Trial allocation strategies of the 2021 NUSTs

Within the Northern Uniform Soybean Trials, the placement and usage of test sites was decided by individual cooperating breeders and almost entirely determined by the practical considerations of availability, cost, and accessibility. When breeders had the opportunity to choose between two or more comparable locations, they sometimes chose sites which were farther from sites already in-use, using distance as a proxy for environmental variation. However, there was no formal assessment of environmental variation within the TPE, and no strategy to distribute testing resources according to environmental representation (Aaron Lorenz, personal communication, March 23, 2023).

By treating AEZ-MGs as a meaningful representation of the underlying variation of the TPE, breeders may strategize the distribution of testing to increase the environmental representation of the testing network and better develop adapted soybean varieties. Breeders may increase the environmental representation of the test network as a whole by distributing sites and testing across unique AEZs, or prioritizing placing new sites and testing in zones without representation (Hyman et al., 2013; Rattalino Edreira et al., 2018). Breeders may also increase the environmental representation of individual variety tests by spatially stratifying testing between AEZs and randomizing placement within them (Hansen and Jones, 2000; Williams et al., 2008; Hyman et al., 2013). The intensity of testing within each AEZ may be weighted by the amount crop cultivated within that area, or by environmental variation of the AEZ, to maintain an appropriate degree of representation (Annicchiarico et al., 2005; Rattalino Edreira et al., 2018).

We applied the AEZ-MG characterization of the soybean TPE to the 2021 Northern Uniform Soybean Trials. We evaluated how testing was distributed within the 2021 season in regard to the elements of this characterization, and how it might be redistributed to improve the environmental representation of the test network.

The testing distribution of the NUSTs was quantified in terms of uniform test replicates. Within our data, each record represents the results of a cultivar as averaged from “three or four replications” within a multiple-row plot (USDA ARS, 2022). Without access to raw data, we used three times the number of records for a location as the number of replicates at that site. The total number of uniform test replicates for sites within a subregion (AEZ, MG, or AEZ-MG) was used as a measurement of testing intensity within that subregion and the ability of the testing network to represent the associated environment. Each replicate represents the resources required to test one replicate, or the resources required to grow and harvest a single soybean plant. Every three (or four) replicates represents the ability for a location to test an additional cultivar within the uniform trials.

We compared two distribution strategies for how the resources of the 2021 NUSTs might be reallocated. The first strategy complements selection for general adaptation: replicates are distributed proportionally to the relative cultivation of the subregion, measured as “crop frequency”, or the frequency at which total recorded soybean cultivation occurred within that subregion. The second strategy complements selection for specific adaptation: replicates are distributed proportionally to the total environmental variation of the subregion. We used Chi-Square Goodness of Fit tests to determine the proportionality of the testing distributions and to make recommendations for how testing should be redistributed.

Of the sites used in 2021, none were located within AEZs 12 or 13. This is likely because AEZs 12 and 13 are the southernmost zones of our characterization, and the locations within them would belong to the Southern Uniform Soybean Tests (SUSTs) (USDA ARS, 2022). For this reason, AEZs 12 and 13 were excluded from any analysis of testing distribution. AEZs 12 and 13 were not considered as possible zones where replicates might be distributed, and the areas of the zones were not included in calculations of cultivation frequency or environmental variation within the TPE.

## 3 Results

### 3.1 Agro-ecological zones

**Figure 2:**
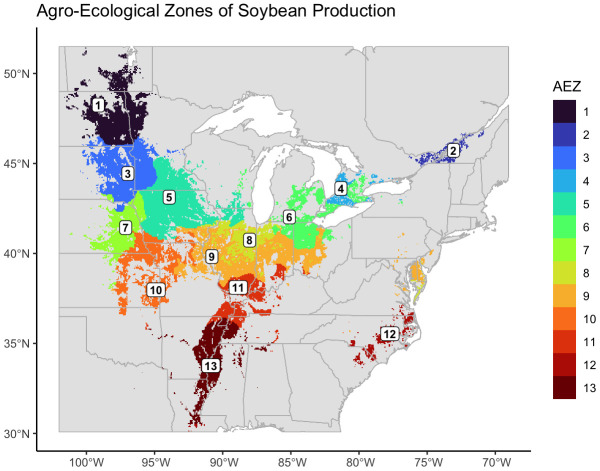
Map of thirteen agro-ecological zones of soybean production in the United States and Canada for the target area of soybean production. AEZs were created by k-means clustering of scaled variables of annual mean temperature, temperature annual range, mean temperature of the wettest quarter, mean temperature of the warmest quarter, mean temperature of the coldest quarter, annual precipitation, wettest quarter precipitation, warmest quarter precipitation, and elevation.

We created thirteen agro-ecological zones (AEZ) of soybean production in the United States and Canada using k-means clustering of scaled bioclimatic data within the soybean growing region and splitting the central cluster by SOC (**Figure 2**). These AEZs explain 86.2% of environmental variation across the TPE.

**Figure 3:**
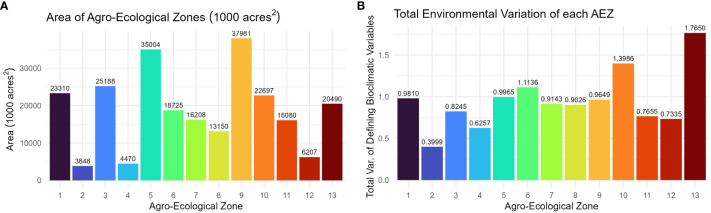
**(A)** Column chart of the area in 1000 acres^2^ of each agro-ecological zone of soybean production in the United States and Canada. **(B)** Column chart of the total environmental variation of the AEZ, defined as its total variation of the scaled bioclimatic variables used to generate the set of AEZs.

The AEZs varied significantly in size (Chi-Square Test of Homogeneity, p< 0.001). The largest AEZs were 9, which forms a horseshoe through Illinois and Indiana, and 5, which covers Iowa and southern Minnesota (**Figure 3A**). The smallest AEZs were 4, which covers a portion of southwest Ontario, 2, which covers the area surrounding the St. Lawrence River in Quebec, and 12, which covers Virginia’s Tidewater region (**Figure 3A**).

The total environmental variation of the AEZ was defined as its total variance of the scaled bioclimatic variables used to generate the set of AEZs. The total environmental variation of the AEZs was proportional to their size (Chi-Square Goodness of Fit Test, p = 0.9943). The AEZs that demonstrated the greatest environmental variation were 10, which covers an area centered on Kansas City, and 13, which covers the southernmost area of the TPE along the Mississippi (**Figure 3B**). The AEZs that demonstrated the least environmental variation were 2, which covers the area around the St. Lawrence River, and 8, which covers northern Indiana and central Illinois (**Figure 3B**).

**Figure 4:**
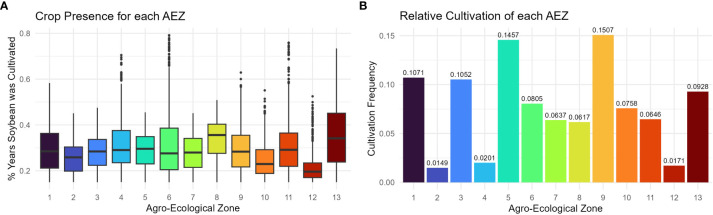
**(A)** Box plot of crop presence for the cells within each agro-ecological zone of soybean production in the United States and Canada. Crop presence for each cell was defined as the percent of years that soybean was grown within the cell of all years recorded. **(B)** Column chart of the relative cultivation of each agro-ecological zone of soybean production in the United States and Canada. The cultivation frequency of each AEZ, the frequency at which total recorded soybean cultivation occurred within an AEZ, was calculated as the sum of crop presence values of each cell within the AEZ divided by the sum of crop presence values across the TPE.

The consistency with which soybean was cultivated within an AEZ was measured as “crop presence.” Crop presence for each cell was defined as the percentage of years that soybean was grown within the cell of all years recorded. Distributions of crop presence were not equal across AEZs (ANOVA, p< 0.001). AEZ 12, covering the Tidewater region, has the lowest mean crop presence and represents the area where soybean is least consistently cultivated (**Figure 4A**). Conversely, AEZ 8, representing fertile soils of the central Midwest, has the highest mean crop presence and represents the area where soybean is most consistently cultivated (**Figure 4A**).

The relative cultivation of the AEZs was quantified as “cultivation frequency”, the frequency at which total recorded soybean cultivation occurred within a particular AEZ (weighted for the difference in record length between Canadian and USA land use data). This statistic was used to determine the proportion of soybean cultivation within the TPE that each AEZ represents. The cultivation frequency of each AEZ was calculated as the sum of crop presence values for each cell within the AEZ divided by the sum of crop presence values for the TPE.

The relative cultivation of the AEZs was proportional to their area (Chi-Square Goodness of Fit Test, p = 0.9999). AEZ 9, which forms a horseshoe through Illinois and Indiana, represents the area with the greatest proportion of soybean cultivation, and AEZ 2, which covers the area surrounding the St. Lawrence River in Quebec, represents the area with the least proportion of soybean cultivation (**Figure 4B**).

### 3.2 Maturity groups

**Figure 5:**
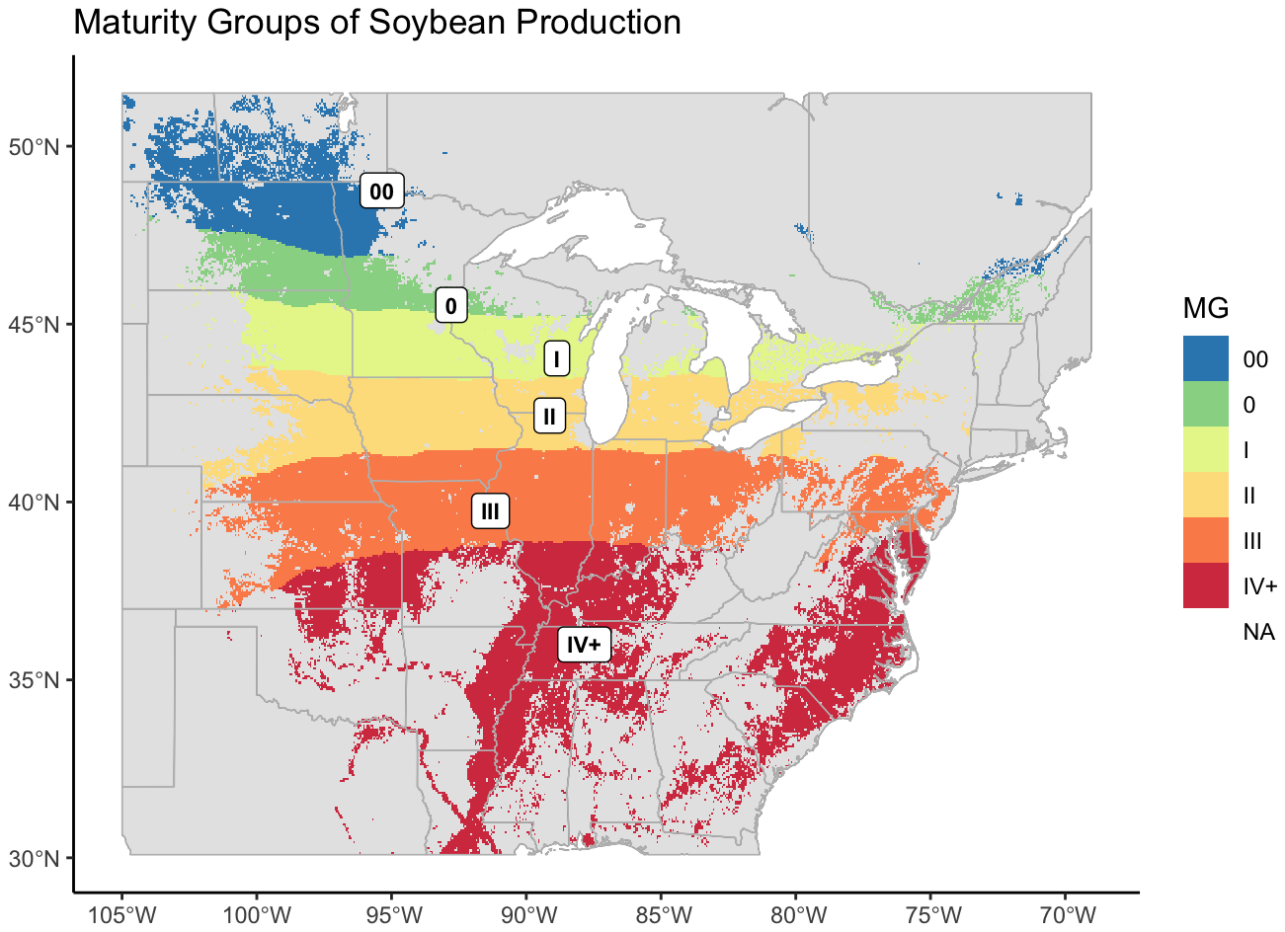
Revised maturity groups for US-Canadian soybean production, mapped over the entire area where soybean was recorded as being grown. US records of soybean cultivation were from 2007 – 2022, Canadian records were from 2009 – 2022.

From our models we created a map of optimum maturity groups across the area of soybean cultivation in the United States and Canada. From far-west to west MG II narrows and the other MGs curve inward to form five regular, horizontal bands across the TPE (**Figure 5**).

Because the map was generated from the designations of check varieties, and the highest maturity check within the NUST system is a late IV, or 4.5, the model did not accurately predict higher maturity values. MGs IV and greater were therefore labeled as IV+.

**Figure 6:**
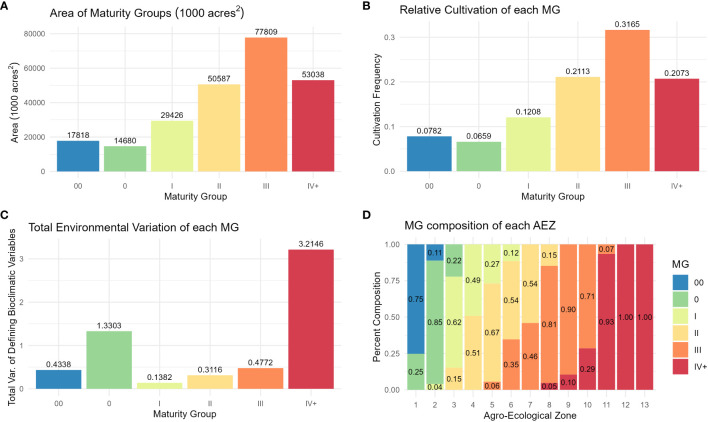
**(A)** Column chart of the area in 1000 acres^2^ of each maturity group. **(B)** Column chart of the relative cultivation of soybean within each maturity group. **(C)** Column chart of the total environmental variation of each maturity group, defined as its total variation of the scaled bioclimatic variables used to generate the set of AEZs. **(D)** Proportional representation of the maturity groups within each agro-ecological zone.

Distributions of crop presence were not equal across MGs (ANOVA, p< 0.001). The largest MG was MG III, and the smallest was MG 0 (**Figure 6A**). As with AEZs, the relative cultivation of the MGs was proportional to their area (Chi-Square Goodness of Fit Test, p = 0.9999). The MGs representing the greatest and least proportions of soybean cultivation in North America were also MG III and 0 respectively (**Figure 6B**). The total environmental variation of MGs was not equal across MGs (ANOVA, p< 0.001), nor was it proportionate to their size (Chi-Square Goodness of Fit Test, p = 0.0191). MG IV+ had the greatest environmental variation of the MGs, and MG I the least (**Figure 6C**). Most MGs overlapped two to three AEZs (**Figure 6D**).

### 3.3 Application to the 2021 NUSTs

**Figure 7:**
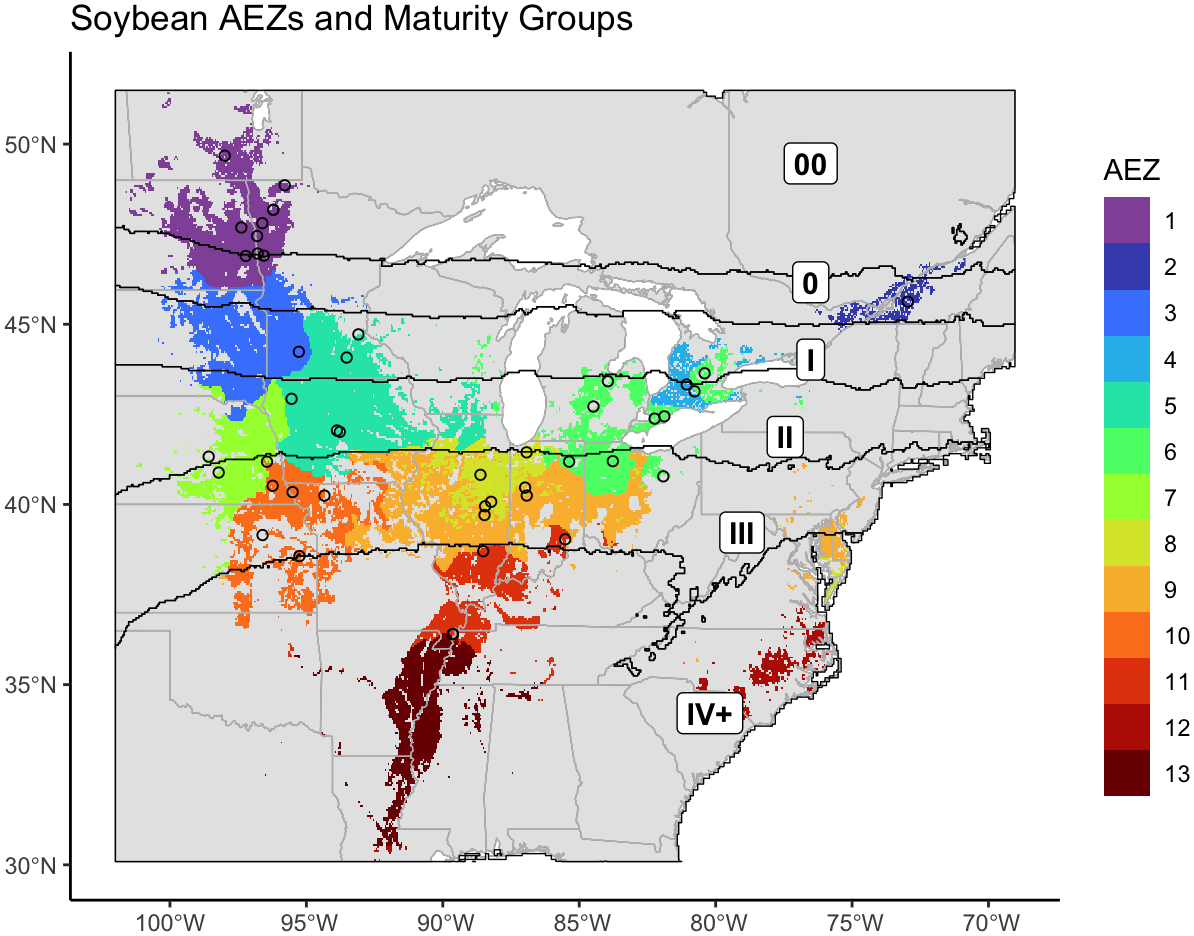
Map of the agro-ecological zones of the target area of soybean production, overlaid with the boundaries of the newly revised maturity groups. The intersections of these two classifications are equivalent to AEZ-MGs. The locations of NUST test sites used in 2021 are marked with circles.

The previously generated AEZs, MGs, and the combined AEZ-MG characterization were applied to the locations of the NUST sites (**Figure 7**). We then assessed the testing of the 2021 NUST in terms of representation of these dimensions of the environment.

We began by evaluating distribution of testing within the 2021 NUSTs in terms of representation to the soybean AEZs, and how it might be redistributed under strategies looking to increase the representation of the environments where soybean is most frequently cultivated or the full environmental variation of the TPE (**Table 1**).

**Table 1:** Recommended testing distribution across AEZs, relative to either cultivation frequency or environmental variation.

AEZ	Observed Replicates	Distribution by Cultivation Frequency		Distribution by Environmental Variation	
		Recommended Number of Replicates	Adjustment to Reach Recommended	Recommended Number of Replicates	Adjustment to Reach Recommended
1	1479	963	-516	794	-685
2	99	134	35	324	225
3	72	946	874	667	595
4	102	180	78	506	404
5	741	1310	569	806	65^†^
6	1359	724	-635	901	-458
7	717	573	-144	740	23^†^
8	729	555	-174	730	1^†^
9	1467	1355	-112	781	-686
10	612	681	69^†^	1132	520
11	624	580	-44^†^	619	-5^†^

^†^Not significant at α = 0.0023 (α = 0.05, two-tailed, Bonferroni correction with eleven comparisons).

Values are in counts of replicates n, and represent the resources required to grow and harvest n soybean plants or to test n replicates/n/3 cultivars. Values in the “Adjustment to Reach Recommended” column highlighted red indicate the recommended removal of replicates, while boxes highlighted blue indicate the recommended addition of replicates. If a value in this column was not highlighted (i.e., has a white background), this indicates that no significant recommendation was made: the difference in the recommended number of replicates was not significant (marked with a †).

The numbers of replicates within the AEZs in 2021 were significantly disproportionate to the frequency of soybean cultivation within the AEZs, and the total environmental variation of the AEZs (Chi-Square Goodness of Fit Test, p< 0.001, p< 0.001). AEZs 1, 6, 8, and 9, are significantly overrepresented under both criteria, and AEZs 2, 3, 4, 5, 10, and 11 are significantly underrepresented (Standardized Residuals from Chi-Square Goodness of Fit Test with Bonferroni Correction, p< 0.0023 for all). AEZ 7 is significantly overrepresented by testing under distribution by cultivation frequency but not under distribution by environmental variation (Standardized Residuals from Chi-Square Goodness of Fit Test with Bonferroni Correction, p< 0.0023, p = 0.2848 respectively).

We next evaluated the representativity of the 2021 NUST testing to our maturity groups, using the same method of analysis and strategies of redistribution (**Table 2**).

**Table 2:** Recommended testing distribution across MGs, relative to either cultivation frequency or environmental variation.

MG	Observed Replicates	Distribution by Cultivation Frequency		Distribution by Environmental Variation	
		Recommended Number of Replicates	Adjustment to Reach Recommended	Recommended Number of Replicates	Adjustment to Reach Recommended
00	1215	703	-512	588	-627
0	363	592	229	1802	1439
I	402	1086	684	187	-215
II	1470	1900	430	422	-1048
III	4143	2845	-1298	647	-3496
IV+	408	875	467	4355	3947

Values are in counts of replicates n, and represent the resources required to grow and harvest n soybean plants or to test n replicates/n/3 cultivars. Values in the “Adjustment to Reach Recommended” column highlighted red indicate the recommended removal of replicates, while boxes highlighted blue indicate the recommended addition of replicates. All adjustments in testing distribution were significant at α = 4.17e-3 (α = 0.05, two-tailed, Bonferroni correction with six comparisons). MG IV+ excludes AEZs 11 and 12.

The numbers of replicates within the MGs in 2021 were significantly disproportionate to the frequency of soybean cultivation within the maturity group and the total environmental variation within the maturity group (Chi-Square Goodness of Fit Test, p< 0.001, p< 0.001). MGs 00 and III were significantly overrepresented by testing relative to their frequency of cultivation, while MGs 0, I, II, and IV+ were significantly underrepresented (Standardized Residuals from Chi-Square Goodness of Fit Test with Bonferroni Correction, p< 0.004 for all). In terms of environmental variation, MGs 00, I, II, and III were significantly overrepresented by testing, while MGs 0 and IV+ were significantly underrepresented (Standardized Residuals from Chi-Square Goodness of Fit Test with Bonferroni Correction, p< 0.004 for all).

Using the intersected AEZ-MG characterization, we gain more specific estimates for how testing might be distributed between the combined AEZ-MG regions (**Table 3**). This characterization can also be used to make recommendations of where test sites should be added or removed from AEZ-MG regions. If no testing currently exists within a region, and a redistribution strategy has a statistically significant suggestion to add test replicates, and this recommendation is large enough to justify at least one test site, then that would be interpreted as a recommendation to establish a test site in that region. Conversely, if a strategy makes a statistically significant suggestion to remove testing from a region and the remaining testing is not large enough to justify running at least one test site, then that would be interpreted as a recommendation to remove all testing from that region. For our purposes, the minimum number of replicates needed to justify operating at least one test site within a region was 54, the fewest number of replicates at any of the NUST sites used in 2021.

**Table 3:** Recommended testing distribution across AEZ-MGs, relative to either cultivation frequency or environmental variation.

AEZ-MG	Observed Replicates	Distribution by Cultivation Frequency		Distribution by Environmental Variation	
		Recommended Number of Replicates	Adjustment to Reach Recommended	Recommended Number of Replicates	Adjustment to Reach Recommended
1 00	1215	692	-523	540	-675
1 0	264	271	7^†^	578	314
2 00	0	11	11	84	84
2 0	99	118	19^†^	155	56
2 I	0	5	5^†^	22	22
3 0	0	203	203	609	609
3 I	72	599	527	198	126
3 II	0	144	144	147	147
4 I	0	74	74	197	197
4 II	102	106	4^†^	144	42
5 I	144	351	207	47	-97
5 II	597	890	293	382	-215
5 III	0	69	69	15	15
6 I	186	57	-129	396	210
6 II	561	371	-190	252	-309
6 III	612	295	-317	43	-569
7 II	210	328	118	814	604
7 III	507	245	-262	346	-161
8 II	0	60	60	31	31
8 III	729	476	-253	254	-475
8 IV+	0	18	18	326	326
9 III	1467	1226	-241	495	-972
9 IV+	0	129	129	719	719
10 III	567	500	-67^†^	459	-108
10 IV+	45	181	136	269	224
11 III	261	34	-227	61	-200
11 IV+	363	547	184	419	56^†^

^†^Not significant at α = 9.62e-4 (α = 0.05, two-tailed, Bonferroni correction with twenty-six comparisons).

Values are in counts of replicates n, and represent the resources required to grow and harvest n soybean plants or to test n replicates/n/3 cultivars. Values in the “Adjustment to Reach Recommended” column highlighted red indicate the recommended removal of replicates, while boxes highlighted blue indicate the recommended addition of replicates. If a value in this column was not highlighted (i.e., has a white background), this indicates that no significant recommendation was made: the difference in the recommended number of replicates was not significant (marked with a †), or a recommended addition to an AEZ-MG without a test site was less than 54 replicates, and therefore below the minimum to justify creating a new test site.

The number of replicates within the AEZ-MGs were disproportionate to both the cultivation frequency and the environmental variation of those regions (Chi-Square Goodness of Fit Test, p< 0.001, p< 0.001). AEZ-MGs 1-00, 6-II, 6-III, 7-III, 8-III, 9-III, and 11-III are significantly overrepresented by testing under both distribution strategies (Standardized Residuals from Chi-Square Goodness of Fit Test with Bonferroni Correction, p< 9.62e-4 for all). AEZ-MG 6-1 is significantly overrepresented by testing only under the strategy of distribution by cultivation frequency, while AEZ-MGs 5-I, 5-II, and 10-III are significantly overrepresented by testing only under the strategy of representation by environmental variation (Standardized Residuals from Chi-Square Goodness of Fit Test with Bonferroni Correction, p< 9.62e-4 for all). The strategy of distribution by cultivation frequency recommends no longer operating test sites within 11-III, while the strategy of distribution by environmental variation recommends no longer operating test sites within 5-I or 6-III, as the recommended number of test replicates within these regions would not be enough to justify operating a test site.

Both strategies find that AEZ-MGs 3-0, 3-I, 3-II, 4-I, 7-II, 9-IV+, and 10-IV+, are significantly underrepresented by testing (Standardized Residuals from Chi-Square Goodness of Fit Test with Bonferroni Correction, p< 9.62e-4 for all). Both strategies also implicitly recommend establishing test sites in 3-0, 3-II, 4-I, and 9-IV+, as no testing currently exists in those AEZ-MGs, and enough testing has been recommended to warrant the creation of new sites. AEZ-MGs 5-I, 5-II, 5-III, 8-II, and 11-IV+ are significantly underrepresented by testing only under the strategy of distribution proportional to cultivation frequency, while 1-0, 2-00, 2-0, 6-I, and 8-IV+ are significantly underrepresented only under the strategy of distribution proportional to environmental variation (Standardized Residuals from Chi-Square Goodness of Fit Test with Bonferroni Correction, p< 9.62e-4 for all). The strategy of distribution by cultivation frequency would recommend establishing an additional new test site in 5-III, while the strategy of distribution by environmental variation would recommend establishing additional new test sites in 2-00 and 8-IV+.

## 4 Discussion

### 4.1 Agro-ecological zones

We created thirteen agro-ecological zones of soybean production in the United States and Canada representing environmental divisions of the area of soybean cultivation. Our final AEZs were largely geographically contiguous (**Figure 2**). The two exceptions were AEZs 6 and 9. AEZ 6 suggests significant climatic variation within the Ontario Peninsula between the land near or on either side of the Niagara and Onondaga escarpments. AEZ 9 suggests the environmental similarity between the central Midwest and the Delmarva peninsula.

Knowledge of noncontiguous AEZs is particularly useful to breeders. Breeders often use the physical proximity of two or more sites as a proxy for their environmental similarity. Noncontiguous AEZs therefore reveal nonintuitive patterns of environmental variation and potential representation. For example: a test site within one portion of a disconnected AEZ would better recreate the environmental conditions of the other portion than a test site which was physically closer to the other portion but was located within a different AEZ.

**Figure 8:**
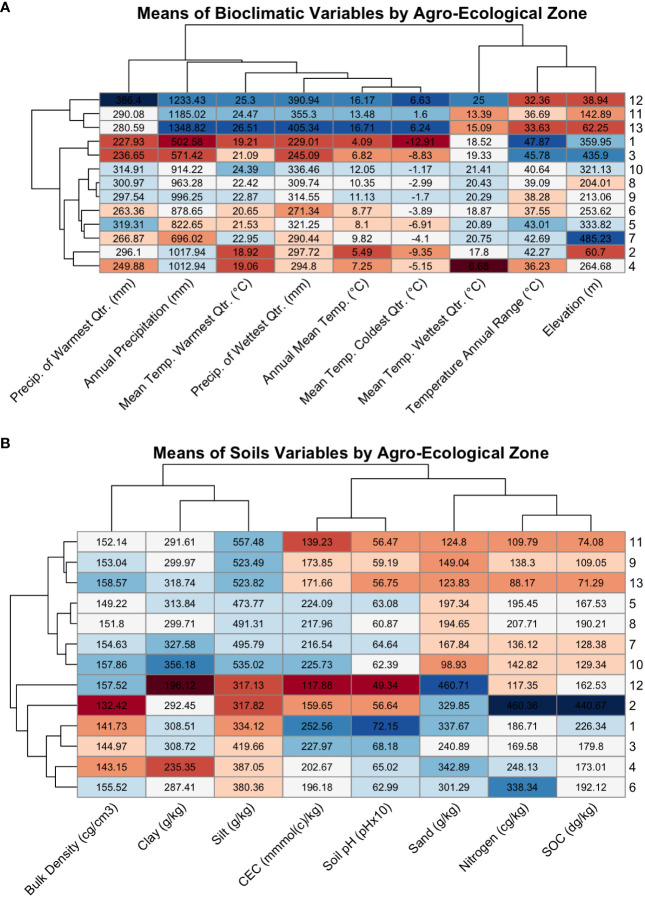
**(A)** Heatmap of the means of the WorldClim bioclimatic variables for each agroecological zone, colored by extremity. Each row contains the means of the bioclimatic variables for an AEZ, with the AEZs indicated by the label on the right side of the plot. The dendrogram on the left of the plot indicates the similarity of the AEZs to each other. Each column contains the mean values of a bioclimatic variable for each of the thirteen AEZs, with the variables indicated by the diagonal labels at the bottom of the plot. The dendrogram above the plot indicates the similarity of the bioclimatic variables to each other. The rows and columns of the plot were arranged by dendrogram structure. Cells were colored by the z-score of the value relative to the scaled bioclimatic variable, or the extremity of that value versus the mean of the bioclimatic variable, with blue indicating positive values and red indicating negative values. **(B)** The same style of heatmap, but comparing the means of soil properties across AEZs.

Most AEZs had the greatest environmental similarity with AEZs they bordered or were of similar latitude to (**Figure 8A**). This aligns with the fact that latitude is a major driver of temperature and precipitation, and that the AEZs represent divisions of a continuous growing environment. The exception to this observation was AEZ 4, which was unexpectedly dissimilar to the noncontiguous 6 which bordered it to the north-east and south-west. Compared to AEZ 6, AEZ 4 had a lower mean temperature in its wettest quarter, greater precipitation in its wettest quarter, and greater precipitation overall.

These differences may be due to the presence of lake-effect snow from Lake Huron and Lake Erie (Liu and Moore, 2004). If so, these effects would not be relevant to soybean adaptation. A possible way to account for irrelevant climatological data in a future iteration of this procedure would be to collect the means of bioclimatic variables only during the expected period of soybean growth, by aggregating either monthly or daily weather data. The trade-off of this decision would be that fewer bioclimatic variables are available on a daily or monthly basis, which could potentially affect the strength of the AEZ clustering and their validity as environments.

As with bioclimatic variables, the soil properties of the AEZs reflect partitioning of continuous patterns of environmental variation. We can observe the division that was made between AEZs 8 and 9: AEZ 8 has greater mean SOC than the latter, as well as higher mean nitrogen content and a higher mean carbon exchange capacity (**Figure 8B**). These properties indicate that soils in AEZ 8 are more productive than soils in AEZ 9. The division that was created between 8 and 9 reflects the meaningful difference in soil fertility that breeders and growers would encounter when growing in that region.

We hypothesized that for a TPE with clinal environmental variation, larger AEZs would capture greater environmental variation and smaller AEZs less. This was supported by our results: the total environmental variation of the AEZs appears to be proportional with size (Chi-Square Goodness of Fit Test, p = 0.9943). We similarly hypothesized that larger AEZs would have greater relative cultivation and smaller AEZs less, as a result of simply containing more area where the crop is grown. This also held true: the relative cultivation of the AEZs also appears to be proportional to their area (Chi-Square Goodness of Fit Test, p = 0.9999). The largest AEZs, 5 and 9, represented the areas with the largest proportions of soybean cultivation, and the smallest AEZs, 2 and 4, represented the areas with the least proportions of soybean cultivation.

### 4.2 Maturity groups

Because the map was generated from the designations of check varieties, and the highest maturity check within the NUST system is a late IV, or 4.5, the maturity map we generated labeled maturity groups IV or greater as IV+ (**Figure 5**). This limitation was mitigated by the fact that past MG IV an ‘optimum’ soybean maturity value becomes more difficult to determine. Soybean farmers in the southern United States commonly plant cultivars with designations of MG III or IV in the zones assigned to MGs V, VI, and VII as part of an early soybean production system (ESPS), using the earlier-maturing varieties to avoid drought stress during the reproductive stages of the plant’s development (Purcell et al., 2003; Heatherly and Elmore, 2004; Heitholt et al., 2005). Soybean farmers in the southern United States may also plant earlier maturing varieties as part of systems of double-cropping (Kyei-Boahen and Zhang, 2006). Within these systems farmers plant and harvest a crop of wheat before planting soybean. Because wheat harvests occur after the optimal range of planting dates for full-season soybean, double-crop soybean is planted later in the season and earlier-maturing varieties are used to compensate (Santos Hansel et al., 2019; Minor and Wiebold, 1998).

Although our intention was to use the NUST data to create an updated map of soybean maturity, the map we created greatly resembles the classic map of soybean maturities published by Scott and Aldrich in 1970 (Scott and Aldrich, 1970). While the modeled map lacks the right-upward curve of the Scott-Aldrich map, maturity groups 00 – IV were nearly identical in breadth and placement. The map therefore also resembles an updated map of optimum soybean maturity published in Zhang et al., 2007, which was similar to the Scott-Aldrich map for MGs 0 – III. Unlike the Scott-Aldrich map, the Zhang map was not speculative, but was generated by kriging data from soybean varietal trials. Compared to the Zhang map, the map generated here again lacks a right-upward curve and has a thinner MG 0, which shifts MG 00 further south (Zhang et al., 2007). In other respects, however, the maps were similar.

The consistency of our map with older maturity classifications conflicts with the updated maturity map generated in Mourtzinis and Conley, 2017. The Mourtzinis & Conley used more, and more recent varietal performance data than the Zhang 2007 map to reassess soybean maturity groups across the United States. Compared to the map generated here, the optimum maturity groups of Mourtzinis & Conley map are less regular and are shifted almost one MG farther north (Mourtzinis and Conley, 2017). Our results suggest that despite recommendations to update optimum maturity groups with changing climates and new varietal data, optimum MGs have remained largely unchanged over time.

### 4.3 Application to the 2021 NUST

The distribution of testing in the 2021 NUSTs was not proportionate to either strategy of representation (cultivation frequency or environmental variation), at any level (AEZ, MG, AEZ-MG) at which the distribution of testing was assessed.

**Figure 9:**
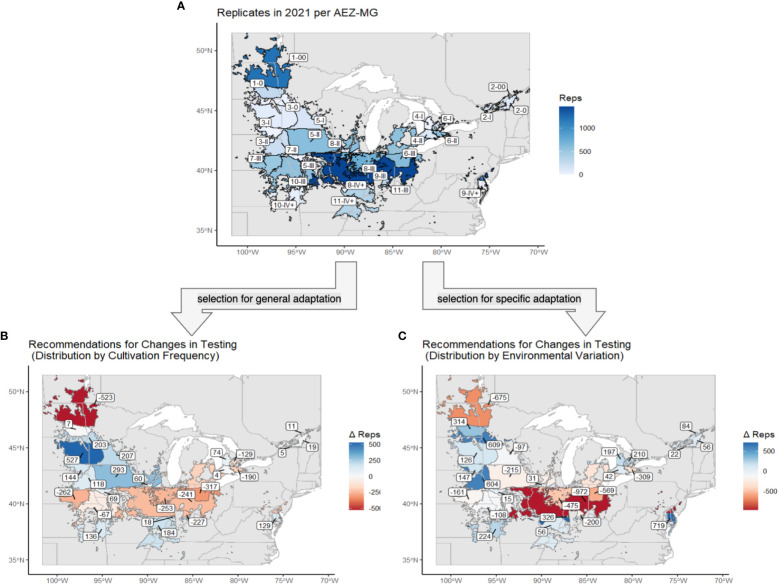
**(A)** The soybean TPE and its AEZ-MG classifications, excluding AEZs 12 and 13. Regions are labeled with their AEZ-MG designation and colored by the number of replicates that were tested at sites within that area. **(B)** The same map of the soybean TPE and its AEZ-MG classifications. Regions are labeled and colored by the change in capacity, in number of replicates, that was recommended under the strategy of distribution by cultivation frequency. **(C)** The same map of the soybean TPE and its AEZ-MG classifications. Regions are labeled and colored by the change in capacity, in the number of replicates, which was recommended under the strategy of distribution by environmental variation.

Both strategies recommended decreasing testing in AEZs 1, 6, and 9 and increasing testing in AEZs 2, 3, and 4 (**Figure 9**). The strategy of representation by cultivation frequency recommended decreasing testing in AEZs 7 and 8 and increasing testing in AEZ 5, while representation by environmental variation recommended increasing testing in AEZ 10. Both strategies overall recommended decreasing testing in MGs 00 and III and increasing testing in MG 0 and IV+. The strategy of representation by cultivation frequency recommended adding testing to MGs I and II, while representation by environmental variation recommended removing it.

The disproportionate amount of testing that that occurred within AEZ 1 and MG 00 under both strategies of representation might be explained by the increasing development of earlier-maturing soybean varieties in Canada in response to warming conditions (Qian et al., 2023).

The overrepresentation of testing in AEZs 9 under both strategies may be influenced by the presence of several university sites, such as those of the University of Illinois at Champaign Urbana and Purdue University, which have the capacity and resources to test a large number of varieties. This effect is more pronounced under the strategy of representation by environmental variation, as this area of the Midwest is largely environmentally homogenous.

AEZs 12 and 13 were excluded from the analysis of testing distribution because the public soybean test sites within those regions were associated with the Southern Uniform Soybean Tests, which were not included in our dataset. AEZs 10 and 11, and MG IV+ are underrepresented under both strategies of representation. Although these regions do contain NUST sites, the underrepresentation of testing within these regions may be similarly affected by the absence of records from SUST sites.

### 4.4 Potential of AEZs for strategizing resource distribution

**Figure 10:**
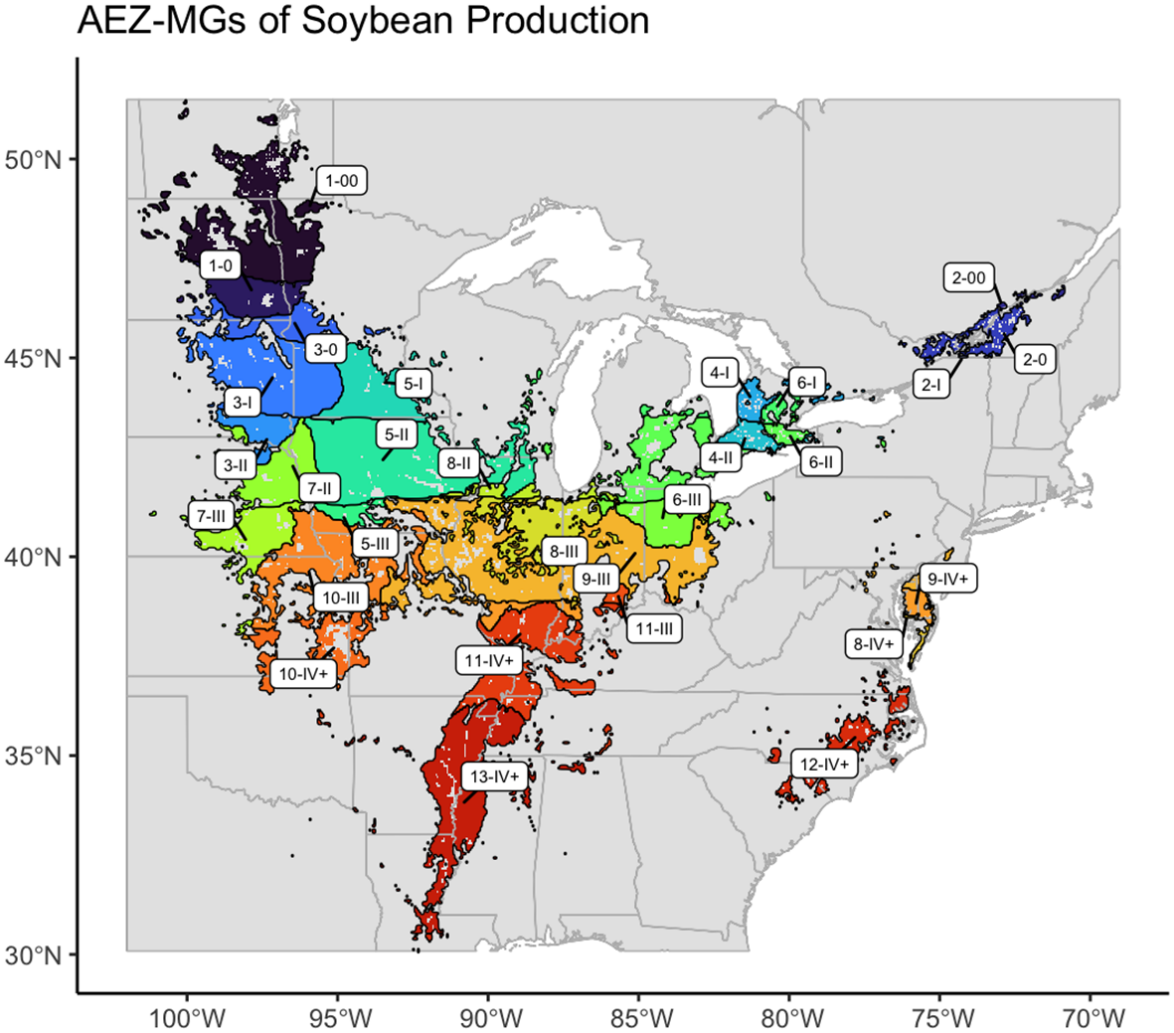
Map of the AEZ-MG characterization of the environment of soybean cultivation in North America.

The final product of this research is an AEZ-MG characterization of the soybean TPE, which may be used to guide the distribution of MET testing in terms of environmental representativity to the area of soybean production in North America (**Figure 10**).

AEZs have several advantages as a method of environmental characterization. The first is that they are meaningful. AEZs are created using bioclimatic variables which capture the annual trends, seasonality, and extreme factors that affect soybean growth and adaptation (Atlin et al., 2000; Hyman et al., 2013). By using AEZs, breeders can directly interpret the effects of particular parameters, such as heat or water availability, on soybean development (Williams et al., 2008; Heinemann et al., 2022). AEZs also have the advantage of stability. AEZs created with the means of long-term climatic data represent the normal conditions of a region and are more likely to reflect the future conditions of the TPE. This makes them useful for informing long-term trial design decisions like the placement of new test sites (Zan et al., 2019).

The AEZs’ basis in agronomically relevant variables and their stability between years together give AEZs the advantage of predictiveness when combined with crop models. Incorporating the AEZ affiliation of MET data as a random genotype x zone effect has been shown to improve the predictive accuracy of genomic selection models (Buntaran et al., 2019, Buntaran, 2021a, Buntaran et al., 2021). Performance predictions derived from models incorporating zonal effects are more accurate, within each AEZ, than predictions derived from general performance over the TPE, and more reliable in accuracy than predictions derived from the results of a single nearest test location (Atlin et al., 2000; Annicchiarico et al., 2005; Piepho and Möhring, 2005; Lado et al., 2016; Neyhart et al., 2021).

Another advantage of AEZs is that they can be defined solely with environmental data. Because AEZs are defined with environmental data that can be interpolated to any location, they have explicit spatial extents within the TPE. This makes it easier to understand the effects of bioclimatic variables within particular AEZs, or to make comparisons between them (Costantini et al., 2016). Being able to link AEZs to specific geographic extents allows breeders to identify miscellaneous environmental pressures that may exist within those regions, such as the presence of specific diseases, differences in soil, or seasonal weather conditions (Caldiz et al., 2002; Williams et al., 2008; Xu, 2016). Predicting genotype performances within AEZs also provides boundaries for where varieties are best adapted and would be recommended (Annicchiarico et al., 2005; Hyman et al., 2013; Bhardwaj et al., 2022).

Within an AEZ system, the environmental affiliation of a location can be determined simply by whether it falls within the boundaries of the AEZ. This presents an advantage over methods like GGE biplot or AMMI analysis, which require specific trial data to determine the classification of a site (Yan et al., 2000). The ability to determine the AEZ affiliation of a site without specific genotype performance data is particularly useful for trial resource distribution because it allows breeders to determine the environmental affiliation of target growing environments or potential new test sites which would otherwise lack the appropriate records (Williams et al., 2008; Hyman et al., 2013; Pérez-Rodríguez et al., 2017; Bhardwaj et al., 2022).

### 4.5 Future directions

This research demonstrated two strategies for how AEZ-MG classifications may be used to guide the distribution of test resources within in MET. These strategies assess the environmental representation of the MET in terms of the proportionate presence of specific environments within the network of test sites. They do not, however, account for representation on a more specific level, such as how replicates were distributed between test sites or where those sites were located.

The proportionate distribution strategies operate as if each of the test sites within the AEZ equally represents the total environment of the AEZ. In reality, the distribution of resources between test sites and the location of those test sites will have their own effects on representation. Each site within an AEZ will represent each point within the AEZ to a greater or lesser extent based on the environmental similarity of those two points. Increasing the number of replicates at a single site may increase the representation of the AEZ-MG environmental class within the MET, but it will not equally increase the environmental representation of each location within the AEZ-MG. Instead, the number of replicates at a single test site will weight the specific representation of that site, to what degree the site has with each point within the AEZ.

The assumption that each site equally represents the total environment of the AEZ prevents this method from evaluating the value of the environmental representation of specific test sites. This information would be useful to inform a decision between one or more new test locations within the same AEZ, or to identify environmentally redundant sites to remove from the trial network. Future research will quantify the value of the representation of each test site within the MET. This information will be used to assess how sites contribute to the trial network’s environmental representation of the TPE, and to select a set of test sites which most efficiently represent its environmental variation.

## 5 Conclusion

The characterization created in this study captures the major components of the environment, spatial climatic variation, and optimum maturity, which influence the range of adaptation of soybean varieties. Breeders may use this characterization to assess the representation of a multi-environment trial of soybean varieties and strategize the distribution of testing resources within the trial, such as the intensity of testing within a particular environment, or the use and disuse of particular test sites, accordingly. By improving the representation of the trial network to the area that it represents, breeders may more efficiently develop adapted soybean varieties to a changing environment.

## Data availability statement

The raw data supporting the conclusions of this article will be made available by the authors, without undue reservation.

## Author contributions

CG: Data curation, Formal analysis, Investigation, Methodology, Software, Visualization, Writing – original draft, Conceptualization. NM: Conceptualization, Funding acquisition, Investigation, Project administration, Resources, Supervision, Writing – review & editing.
